# Salinity and Nitrogen Availability Affect Growth, Oxalate Metabolism, and Nutritional Quality in Red Orache Baby Greens

**DOI:** 10.3390/plants14213292

**Published:** 2025-10-28

**Authors:** Martina Puccinelli, Simone Cuccagna, Rita Maggini, Giulia Carmassi, Alberto Pardossi, Alice Trivellini

**Affiliations:** Department of Agriculture, Food and Environment, University of Pisa, Via del Borghetto 80, 56124 Pisa, Italy; s.cuccagna@studenti.unipi.it (S.C.); rita.maggini@unipi.it (R.M.); giulia.carmassi@unipi.it (G.C.); alberto.pardossi@unipi.it (A.P.); alice.trivellini@unipi.it (A.T.)

**Keywords:** antioxidants, ascorbic acid, halophytes, hydroponics, NaCl, oxalate oxidase

## Abstract

As freshwater resources become increasingly scarce, seawater and brackish water represent alternative sources for crop irrigation, particularly in systems such as saltwater aquaponics. Red orache (*Atriplex hortensis* var. *rubra*) is a halophyte with high antioxidant content but also accumulates antinutrients like nitrate (NO_3_^−^) and oxalate. Oxalate helps plants cope with salinity stress but can cause health issues in humans. This study examined the growth of red orache baby greens in saline and nitrogen-limited hydroponic solutions to assess its adaptability and nutritional quality, focusing on the impact of salinity and reduced nitrogen on antinutrient levels. Four nutrient solutions differing in NaCl (0 or 428 mM) and NO_3_^−^ (10 or 1 mM) were tested. Salinity significantly reduced red orache yield (by 75.5%), pigment levels, antioxidants, and nutrient uptake, while increasing leaf Na and oxalate concentration, ethylene production, and succulence. Salinity decreased NO_3_^−^ concentration and oxalate oxidase (OxO) activity but boosted total ascorbic acid and oxalate accumulation. Low NO_3_^−^ mildly reduced yield (by 25.7%), leaf area, and NO_3_^−^ concentration in leaves, but had no effect on leaf moisture content, succulence, antioxidant capacity, and the concentration of antioxidants, pigments, and total oxalate. In addition, low NO_3_^−^ increased OxO activity, only under non-saline conditions. The high salinity typical of aquaculture effluents strongly reduced red orache baby greens yield and quality to a greater extent than low NO_3_^−^ levels. Both salinity and low NO_3_^−^ reduced NO_3_^−^ concentration in leaves, while salinity increased oxalate concentration, probably due to the reduced activity of OxO.

## 1. Introduction

As freshwater resources become scarce, brackish and highly saline waters, such as seawater, offer an alternative for crop irrigation after desalination or dilution with freshwater [1]. One specific application of seawater in agriculture is saltwater aquaponics, which utilizes water with salinities as high as 35 g L^−1^ (the salinity of seawater) [2]. Aquaponics is a technique that combines the hydroponic cultivation of crop plants with intensive fish farming. Growing plants in aquaponics presents different challenges compared to standard hydroponics. In aquaponic systems, fish, microorganisms, and plants have distinct water quality requirements. Water quality deterioration is mainly influenced by fish feeding rate, density, and feed composition. Consequently, macro- and microelement concentrations in aquaponic solutions are often below optimal levels for hydroponics, leading to plant nutrient deficiencies [3]. In marine aquaponic systems, crops must also tolerate high salinity (up to 35 g L^−1^ NaCl) and nutrient solutions that are relatively poor in nitrogen (1–2 mM NO_3_^−^) but rich in Mg, B, Na, and Cl compared to standard hydroponic formulations [3].

Orache (*Atriplex hortensis*), a member of the Amaranthaceae family, is a facultative halophyte naturally found in arid and semi-arid regions due to its ability to tolerate drought and salinity [4]. It can be consumed fresh or cooked, like spinach (*Spinacia oleracea* L.). Since ancient times, orache has been cultivated as a minor vegetable across Eurasia, from Central Asia to the Mediterranean, where it was originally domesticated. Today, its cultivation is expanding to other temperate and subtropical regions around the world, thanks to its salt tolerance and impressive nutritional profile, including its high-quality protein leaf and seed content [5]. Red orache (RO, *Atriplex hortensis* var. *rubra*) is distinguished by its leaves that are rich in betalains, which give them a red-purple color [6]. These compounds are typical of Amaranthaceae and have significant antioxidant properties [7], often exceeding those of anthocyanins [6]. Red orache can be utilized like other red-leafed plants, particularly as baby greens in mono- or mixed-species salads; these products can command high prices on the market [8]. Baby greens are immature and tender fresh products with higher antioxidant properties than mature plants of the same species [9].

Despite its beneficial nutritional profile, RO accumulates antinutrients such as nitrate (NO_3_^−^) and oxalate like other species in the Amaranthaceae [10]. Oxalic acid is naturally present in plants, with concentrations ranging from 3% to 80% of DW depending on genotype, organ, and growing conditions [11]. In plant tissues, it occurs both as free oxalic acid (H_2_C_2_O_4_) and as oxalate salts, forming insoluble complexes with Ca or Mg and soluble salts with Na or K [11]. The term “oxalate” refers to the deprotonated anion (C_2_O_4_^2−^), which readily binds metal cations, whereas oxalic acid denotes the free, protonated form. Oxalic acid metabolism contributes to plant responses to abiotic and biotic stresses through ROS regulation, heavy metal detoxification, secondary metabolite production, hormonal interactions, and pathogen defense [11].

Oxalate can adversely affect human health [12]; when taken in excess, oxalate can bind to minerals such as Ca, forming insoluble crystals in the body and leading to kidney stones. Furthermore, soluble oxalate can interfere with the absorption of Ca, Mg, and Fe from food, leading to mineral deficiencies [13]. In plants, oxalic acid is an organic acid present in some plants in soluble and insoluble oxalate [14]. The synthesis of oxalate in plants is closely related to maintaining ionic balance between inorganic cations (K^+^, Na^+^, NH_4_^+^, Ca^2+^, Mg^2+^) and anions (NO_3_^−^, Cl^−^, H_2_PO_4_^−^, SO_4_^2−^) [15]. Oxalate can help the plant’s tolerance to abiotic stresses, such as salinity stress. For instance, oxalate accumulation in vacuoles is pivotal for maintaining ion balance under salinity stress [16].

Oxalate metabolism in plants comprises both biosynthetic and degradative pathways, which are still not fully elucidated. Oxalate can arise from several metabolic routes, including glycolate and ascorbate catabolism [11], while its degradation is mainly mediated by enzymes such as oxalate oxidase (OxO), oxalate decarboxylase, and oxalyl-CoA synthetase [17]. Understanding the balance between these processes is essential for interpreting oxalate accumulation under varying nutritional or environmental conditions. Oxalate oxidase stands as a pivotal enzyme in plants, not only catalyzing the oxidation of oxalate to carbon dioxide and hydrogen peroxide but also playing multiple roles in plant physiology and defense mechanisms [11]. The levels of NO_3_^−^ and ammonium (NH_4_^+^) in the growing medium are known to influence oxalate production in plant tissues [18]. Nitrate application is more likely to be responsible for oxalate buildup than NH_4_^+^ application as NO_3_^−^ increases the activity of NO_3_^−^ reductase and glutamine synthetase in leaves, thus promoting cation accumulation in plants and stimulating oxalate biosynthesis for intracellular pH homeostasis [11]. Additionally, NO_3_^−^ may inhibit OxO activity by binding to its active site, reducing oxalate degradation [19].

This study aimed to evaluate the adaptability of RO to hydroponic baby green production under conditions of high salinity and reduced nitrogen availability, focusing particularly on their combined effects on leaf nutritional quality and antinutrient accumulation (NO_3_^−^ and oxalate). Plants were grown in a floating raft system using nutrient solutions with different concentrations of NaCl (0 and 428 mM; 25 g L^−1^) and NO_3_^−^ (10 and 1 mM) in a factorial design. The control represented standard hydroponic conditions, while the saline–low-nitrogen treatment simulated saltwater aquaponic environments. By linking plant performance and nutritional traits to combined salinity and nitrogen stress, this study provides new insights into the potential of RO as a resilient crop for saline or resource-limited soilless systems.

## 2. Results

Since ANOVA revealed no significant interaction between NaCl and NO_3_^−^ concentration for almost all measured parameters, the results are reported and discussed focusing solely on the main effects of these two factors for clarity and brevity.

### 2.1. Effects of NaCl Salinity

**Figure 1:**
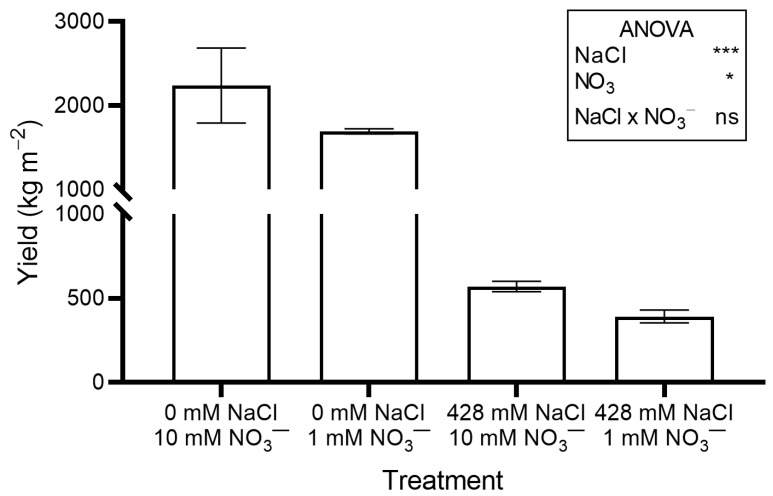
Yield (shoot fresh weight) of red orache (*Atriplex hortensis* var. *rubra*) plants grown hydroponically for 17 days with different nutrient solutions, varying in the concentration of NaCl and NO_3_^−^. Mean values (±SE) of three replicates. Significance: *** *p* ≤ 0.001; * *p* ≤ 0.05; ns = not significant.

The use of a saline nutrient solution markedly reduced crop yield (Figure 1), leaf fresh weight (FW), leaf dry weight (DW), stem DW, root DW, total DW, and leaf area index (LAI) (Table 1), compared to the non-saline solution. The relative growth rate (RGR), leaf area ratio (LAR), and specific leaf area (SLA) were also reduced in salinized plants, while net assimilation rate (NAR) increased (Table S1).

Leaf moisture content slightly decreased in salinized plants, while leaf succulence markedly increased in response to NaCl (Table 1).

The concentrations of total phenols, flavonoids, ascorbic acid and antioxidant capacity measured by Ferric Reducing Antioxidant Power (FRAP) and 2,2-difenil-1-picrilidrazile (DPPH) assays were reduced in salinized plants compared to the controls (Table 2). In contrast, NaCl salinity did not affect the total ascorbic acid concentration (Table 2).

Salinity also significantly reduced leaf concentrations of total chlorophylls, carotenoids, and betalains, resulting in lighter leaf color as indicated by higher lightness (Table 3). No significant effects of salinity were observed on other color properties (Table 3).

**Figure 2:**
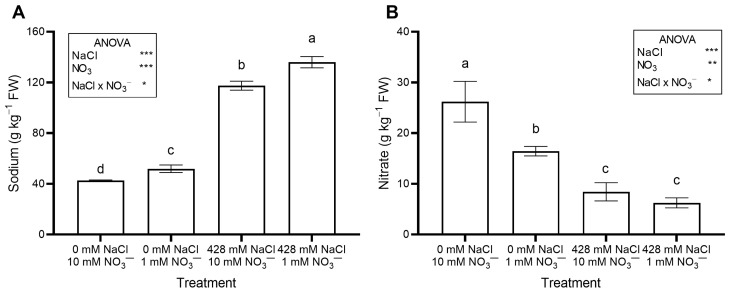
Leaf concentration of Na (**A**) and NO_3_^−^ (**B**) in red orache (*Atriplex hortensis* var. *rubra*) plants grown hydroponically for 17 days with different nutrient solutions, varying in the concentration of NaCl and NO_3_^−^. Mean values (n = 3; ±SE) flanked by the same letter are not statistically different at 5% level after Tukey’s post hoc test. Significance: *** *p* ≤ 0.001; ** *p* ≤ 0.01; * *p* ≤ 0.05; ns = not significant.

**Figure 3:**
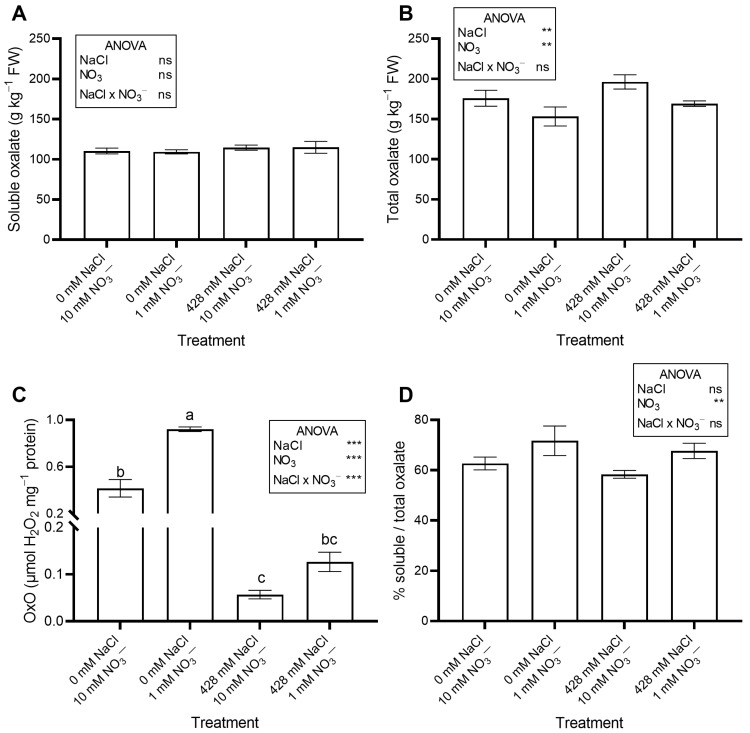
Leaf concentration of soluble (**A**) and total oxalate (**B**), and oxalate oxidase (OxO) activity (**C**) of red orache (*Atriplex hortensis* var. *rubra*) plants grown hydroponically for 17 days with different nutrient solutions, varying in the concentration of NaCl and NO_3_^−^. The percent ratio between soluble and total oxalate is also shown (**D**). In (**C**), mean values (n = 3; ±SE) flanked by the same letter are not statistically different at 5% level after Tukey’s post hoc test. *** *p* ≤ 0.001; ** *p* ≤ 0.01; ns = not significant.

The saline treatments significantly increased leaf Na concentration (Figure 2A) and total oxalate (Figure 3B), while decreasing leaf NO_3_^−^ concentration (Figure 2B) and OxO activity (Figure 3C). The percent ratio between soluble and total oxalate was not significantly affected by salinity, averaging a constant value across treatments (Figure 3D).

**Figure 4:**
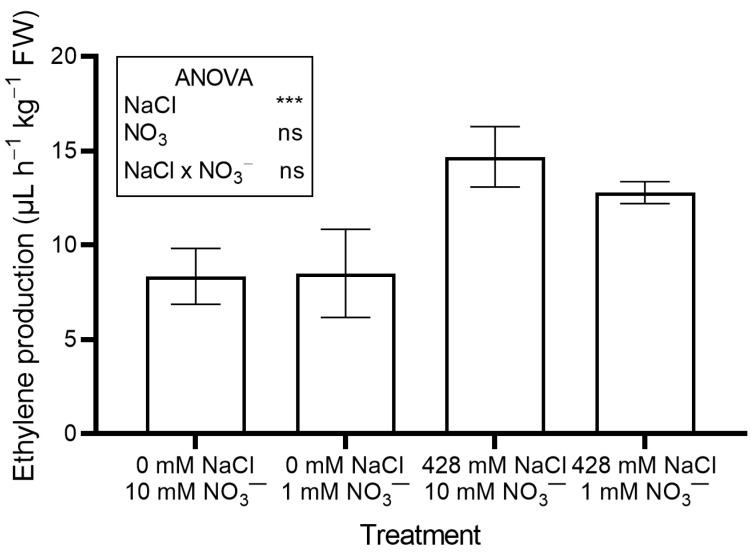
Leaf ethylene production in red orache (*Atriplex hortensis* var. *rubra*) plants grown hydroponically for 17 days with different nutrient solutions, varying in the concentration of NaCl and NO_3_^−^. Significance: *** *p* ≤ 0.001; ns = not significant.

Ethylene production (Figure 4) and leaf performance index (PI) (Figure S1) also rose significantly in salinized plants compared to controls.

The leaf concentrations of N, P, K, Ca, Mg, and Mn were lower in salinized plants than in controls (Table S2), while Fe, Zn, and Cu were unaffected (Table S2).

### 2.2. Effects of Nitrate Concentration

Reducing NO_3_^−^ concentration in the nutrient solution inhibited plant growth, although the effects were less severe than those of NaCl salinity. Crop yield (Figure 1), leaf FW, leaf DW, total DW, and LAI (Table 1) were significantly lower in plants grown with 1 mM NO_3_^−^ compared to those grown with the standard concentration. No significant differences were observed for stem and root DW (Table 1).

The RGR, NAR, and SLA were not significantly affected by NO_3_^−^ level, while a slight but significant reduction in LAR and LWR was observed in plants grown with 1 mM NO_3_^−^ (Table S1).

No significant effects of NO_3_^−^ level were observed on leaf moisture content, succulence (Table 1), antioxidant capacity, total phenols, flavonoids, ascorbic acid (Table 2), pigments (Table 3), soluble oxalate (Figure 3A), color properties (Table 3), ethylene evolution (Figure 4), or PI (Figure S1). In contrast, plants supplied with 1 mM NO_3_^−^ showed a lower leaf concentration of NO_3_^−^ (Figure 2B) and total oxalate (Figure 3B), and a higher Na concentration (Figure 2A). The percent ratio between soluble and total oxalate significantly increased under low NO_3_^−^ conditions (Figure 3D). Reduced NO_3_^−^ supply markedly increased OxO activity, especially under non-saline conditions (Figure 3C).

The leaf concentrations of N, P, K, Mg, and Mn were not significantly influenced by NO_3_^−^ level. However, compared to plants grown with standard NO_3_^−^, those supplied with reduced NO_3_^−^ showed higher Mn and Fe but lower Ca levels (Table S2). Leaf Na concentration was slightly higher in plants grown with 1 mM NO_3_^−^, regardless of NaCl level (Table S2).

## 3. Discussion

This study evaluated the adaptability of RO to hydroponic cultivation of baby greens under contrasting salinity and nitrogen conditions. Using a floating raft system, plants were exposed to nutrient solutions differing in NaCl and NO_3_^−^ concentrations to reproduce the chemical environment typical of saltwater aquaponics. The effects on growth performance, pigment composition, antioxidant capacity, and the accumulation of antinutrients such as NO_3_^−^ and oxalate were analyzed. The following discussion integrates these results to elucidate the physiological mechanisms underlying red orache responses to combined salinity and nitrogen stress, highlighting their implications for sustainable soilless cultivation in saline environments.

### 3.1. Adaptation of Red Orache to Hydroponic Cultivation

In this study, RO adapted well to hydroponic cultivation with a standard nutrient solution. The baby greens yield recorded 17 days after transplanting (DAP; 29 days after sowing, Figure 1) were similar to those reported for red amaranth (*Amaranthus tricolor* L.), a species in the Amaranthaceae closely related to RO, which was grown hydroponically for 26 days in a growth chamber under comparable temperature and light conditions [20].

In our work, the use of saline nutrient solution markedly inhibited crop growth and yield regardless of NO_3_^−^ supply (Table 1 and Figure 1). In a previous study, the growth of RO plants grown in soil was markedly reduced by irrigation with saline water (5 to 15 g L^−1^ of NaCl) compared to those irrigated with NaCl-free water [21]. In contrast, no significant difference in DW was observed in RO and other *Atriplex* species grown hydroponically for 35 days under 0 and 360 mM NaCl salinity, and plant FW was even higher in NaCl-treated plants [22].

The leaf concentrations of N, P, K, Ca, Mg, and Mn were significantly decreased by high salinity (Table S2), consistent with previous results [22]. The antagonism between Na^+^ and other cations [23], and between Cl^−^ and NO_3_^−^ [24] likely accounted for the reduction in K, Ca, Mg and N uptake in plants grown with NaCl-enriched solution. However, the growth inhibition caused by high NaCl in RO plants was not attributable to leaf mineral deficiency, since no visible symptoms of salt toxicity (e.g., leaf scorch) or nutrient deficiency were observed. Leaf concentrations of all essential elements, except Ca, remained within the sufficiency ranges previously reported for related species such as table beet (*Beta vulgaris* subsp. *vulgaris* L.) and spinach (Table S3).

Growth analysis and the analysis of chlorophyll fluorescence transients (JIP test), indicate that differences in biomass production (Figure 1 and Table 1) and RGR (Table S1) between the two salinity treatments were not due to impaired leaf photosynthesis, since both NAR (Table S1) and PI (Figure S1) were higher in salinized than non-salinized plants. Similarly, Calone et al. [22] reported that NaCl salinity did not significantly affect leaf gas exchange or chlorophyll fluorescence parameters in RO and other *Atriplex* species.

In our work, NaCl-induced growth suppression was mainly attributable to a decrease in LAR, a component of RGR (RGR = NAR × LAR). SLA and LWR are the two components of LAR (LAR = SLA × LWR). SLA was significantly reduced under salinity, while LWR was unaffected (Table S1). This indicates that growth inhibition resulted from reduced leaf expansion per unit leaf DW rather than altered dry matter partitioning between leaves and other organs.

The reduction in leaf expansion is an adaptive response to salt stress, which leads to lower transpiration [25]. In our study, reduced leaf expansion in salt-treated plants was likely caused by osmotic stress [25], consistent with the observed decrease in leaf moisture content (Table 1).

Salt-induced stress was further confirmed by increased ethylene production (Figure 4). Salinity stress is known to trigger ethylene production [26]. Ethylene helps maintain Na^+^/K^+^ homeostasis, regulates mineral uptake (including NO_3_^−^), and activates antioxidant defenses [27]. It also interacts with other phytohormones such as abscisic acid, auxins, and jasmonic acid, to coordinate a broad stress response [27].

Reducing the NO_3_^−^ concentration in the nutrient solution decreased crop growth and yield to a lesser extent than high NaCl salinity (Table 1 and Figure 1). Growth analysis and JIP test results suggest that differences in biomass (Figure 1 and Table 1) between the two NO_3_^−^ treatments were not related to photosynthesis. This aligns with the lack of differences in leaf N content (Table S2) [28].

Growth suppression in plants fed with 1 mM NO_3_^−^ was associated with reduced LAR, mainly due to lower SLA, whereas LWR was unaffected (Table S1). This suggests that growth inhibition resulted from smaller leaf area per unit mass rather than altered biomass allocation. In contrast, it was reported [29], that under N limitation, reduced leaf area was primarily due to decreased LWR rather than SLA. Measurements of growth traits (Table 2 and Table S2), leaf N (Table S2), and PI (Figure S1) in RO suggest that, under N limitation, RO maintained leaf N content but reduced leaf area, similar to other species such as sunflower (*Helianthus annuus* L.), maize (*Zea mays* L.) [30] and potato (*Solanum tuberosum* L.) [31]. In RO, high NaCl reduced leaf moisture content while increasing succulence (Table 1). Thus, harvested leaves from saline-grown plants may have improved shelf life compared to non-salinized plants. Increased succulence is a typical halophyte response to salt stress [32], which may positively affect product quality by altering texture [33].

The health benefits of leafy vegetables are largely attributed to antioxidants such as pigments, phenols, flavonoids, and ascorbic acid [34]. In this study, high NaCl reduced antioxidant capacity and concentrations of chlorophylls, carotenoids, betalains, and flavonoids, whereas NO_3_^−^ supply had no effect on these traits (Table 2), nor on leaf moisture or succulence (Table 1).

The lower concentrations of pigments in salinized leaves accounted for their higher lightness compared to non-salinized leaves (Table 3).

Some results were expected. For example, leaf concentrations of chlorophylls and carotenoids generally decrease under salt stress [35]. However, the effect of salt stress on antioxidant compounds varies among Amaranthaceae halophytes. In *Beta vulgaris* spp. *maritima* [36], *Chenopodium quinoa* [37], *Salicornia ramosissima* [38], and *Suaeda maritima* [39], salinity increased phenol and flavonoid concentrations, whereas decreases were observed in *S. europaea* [40] and *S. ramosissima* [39], and in the facultative halophyte *Portulaca oleracea* L. [41].

The reduction in antioxidant compounds in RO under salinity may be explained by their use in scavenging ROS to protect against oxidative damage [42]. In our experiment, only total ascorbic acid slightly increased under 428 mM NaCl compared to 0 mM, likely due to a concentration effect.

### 3.2. Effect of Salinity and Nitrogen Nutrition on Leaf Concentration of Sodium and Antinutrients

Leaf Na concentration increased when the plants were irrigated with a saline nutrient solution (Figure 2A), as expected, due to the much higher Na concentration compared to the control solution. These results agree with previous studies on Swiss chard (*Beta vulgaris* var. *cicla*) [43], sea beet (*Beta vulgaris* subsp. *maritima*) [35,36,43], and spinach [44] grown with different NaCl concentrations in hydroponics.

A large Na intake raises cardiovascular risk, and in adults, the recommended maximum daily intake is 2 g per day [45]. In the current work, leaf Na concentration reached 12.47 g kg^−1^ FW in salinized RO grown at 1 mM NO_3_^−^ (Figure 2A). Thus, consuming a 100 g serving of RO baby greens is safe, as it would provide much less Na than the daily intake limit.

The interaction between NaCl salinity and nitrogen nutrition and metabolism differs significantly between glycophytes and halophytes. In glycophytes, NaCl salinity can reduce the uptake and assimilation of NO_3_^−^ through several mechanisms, such as specific inhibition of NO_3_^−^ transport at the root plasma membrane, a reduced demand for N due to salinity-induced growth restriction, and direct inhibition of key enzymes involved in NO_3_^−^ assimilation, such as nitrate reductase (NR) and glutamine synthetase (GS) [46]. On the other hand, optimal nitrogen fertilization can improve plant’s tolerance to salt stress by regulating antioxidant enzyme activities, as found in maize [47]. In contrast, in halophytes such as *Suaeda physophora* [48], *Suaeda salsa* [49], and *Salicornia europaea* [50], NO_3_^−^ uptake and assimilation are enhanced under saline conditions.

In this study, there was no significant interaction between NaCl and NO_3_^−^ levels in the growing medium with respect to leaf NO_3_^−^ concentration (Figure 2B) and total N (Table S2), both of which were significantly reduced by high salinity and low NO_3_^−^ supply.

Nitrate may negatively affect human health, and because leafy vegetables are among the primary dietary sources, the European Union (EU) has imposed limits for some leafy species such as lettuce, spinach, and rocket [51]. In this work, conducted in autumn, NO_3_^−^ levels in RO were consistently well below the EU maximum for spinach (3.5 g kg^−1^ FW); the highest level measured was 1.70 g kg^−1^ FW in control plants (Figure 2B). Artificial lighting likely limited NO_3_^−^ accumulation in RO leaves, as leaf NO_3_^−^ levels generally decrease with increasing photosynthetically active radiation (PAR), which promotes NO_3_^−^ assimilation [24].

Although no official guidelines exist, adults are generally advised to limit oxalate intake to ~200 mg day^−1^ to prevent kidney stones [52]. In this work, leaf soluble oxalate ranged from 7.19 to 10.57 g kg^−1^ FW. Thus, consuming just 19–28 g of RO leaves would reach the daily limit.

Oxalic acid also impacts vegetable organoleptic traits, particularly causing the phenomenon known as “spinach teeth”, a gritty or chalky sensation caused by calcium oxalate crystal formation in saliva [13].

In our study, RO accumulated higher levels of oxalate (159.9–196.2 g kg^−1^ DW) than *Atriplex halimus* (76.7–106.7 g kg^−1^ DW) and *A. nummularia* (72.9–78.0 g kg^−1^ DW) [53]. At 17 DAP (Figure 3), soluble and total oxalate concentrations increased significantly in NaCl-treated plants, while OxO activity decreased.

Similar trends have been reported in halophytes such as *Suaeda glauca* [54], *Portulaca oleracea* [16], and *Beta vulgaris* ssp. *maritima* [36] grown under up to 400 mM NaCl, where oxalate accumulation contributed to osmotic adjustment and ionic balance between excess cations (Na^+^, K^+^) and anions (Cl^−^, SO_4_^2−^).

Our results are partly consistent with the biochemical model that attributes oxalate accumulation to both de novo synthesis and limited degradation by OxO, which catalyzes oxidation of oxalate to CO_2_ and H_2_O_2_ [11]. Although oxalate breakdown is mediated by OxO, oxalate decarboxylase, and oxalyl-CoA synthase [11], we focused on OxO because our results directly relate to its activity. Since OxO alone only partially accounts for the observed changes in oxalate, further studies are needed to elucidate the contributions of other degrading enzymes.

The metabolism of ascorbate and oxalate are closely interconnected, as ascorbic acid is linked to mitochondrial and photosynthetic electron transport, influencing both production and conversion to oxalate [55]. Oxalate formation from ascorbic acid degradation has been reported in several species with structural, physiological, and biochemical roles [55]. In our experiment, total oxalate (Figure 3) increased, while ascorbic acid decreased (Table 1) in salinized plants and NO_3_^−^ had no effect (Table 2). This decrease in ascorbic acid concentration might be related to its utilization as a substrate for oxalate synthesis.

Evidence indicates a relationship between N nutrition and oxalate biosynthesis [56,57]. Nitrate assimilation into amino acids requires reduction to NH_4_^+^, a process that alkalinizes the cytosol (one OH^−^ released per NO_3_^−^ reduced; [58]). To maintain cytosolic pH within an optimal range, plants synthesize organic acids such as oxalic acid, which release H^+^ ions and help counteract the alkalinization, leading to increased oxalate accumulation. In hydroponic spinach, soluble and total oxalate increased with NO_3_^−^ concentration (up to a certain point) and decreased with the NO_3_^−^/NH_4_^+^ ratio. Similar results were reported in *Atriplex nummularia* [56].

As far as we know, no study has investigated N effects on oxalate accumulation in RO. In our work, low NO_3_^−^ concentration had minor or insignificant effects on leaf oxalate at 17 DAP (Figure 3B). Indeed, in plants grown with 1 mM NO_3_^−^, total oxalate decreased slightly but significantly, while soluble oxalate was unaffected. In contrast, OxO activity increased significantly under 1 mM NO_3_^−^. According to our results, Çalişkan [15] hypothesized that NO_3_^−^ inhibits OxO, promoting oxalate accumulation. Meeuse et al. [59] also found that NO_3_^−^ inhibits oxalate breakdown by OxO in beet extracts. Thus, the reduction in total oxalate concentration observed in our experiment may have been partly driven by enhanced OxO activity under low NO_3_^−^ conditions. Oxalate oxidase plays a central role in oxalate metabolism, catalyzing the oxidative degradation of oxalate into CO_2_ and H_2_O_2_. Its activity is essential for maintaining oxalate homeostasis and preventing excessive oxalate accumulation, which can lead to calcium oxalate crystal formation and reduced calcium bioavailability [60]. However, the relative contribution of OxO compared to other oxalate-degrading enzymes, such as oxalate decarboxylase and oxalyl-CoA synthetase, likely varies among species and environmental conditions [17]. Since the activity of these additional enzymes was not analyzed in this study, a complete understanding of the regulatory mechanisms requires further investigation. Hence, adjusting N levels and/or the NO_3_^−^/NH_4_^+^ ratio in hydroponics could lower leaf oxalate, benefiting human health, as shown by Fontana et al. [61] and Zhang et al. [57], although this strategy may also reduce biomass. Zhang et al. [57] suggested that a NO_3_^−^/NH_4_^+^ ratio of 1 can reduce oxalate without impairing growth. Moreover, blanching effectively removes oxalate, as reported for *Portulaca oleracea* [62] and *Tetragonia expansa* [63]. However, blanching is unsuitable for tender products like baby greens, as it causes wilting and nutrient loss [64].

## 4. Materials and Methods

### 4.1. Plant Material and Growing Conditions

Seeds were purchased from “De Bolster Organic Seeds” (https://www.bolster.eu/, accessed on 15 July 2023), sown into stonewool cubes and kept at 25 °C in a growth chamber until emergence. Twelve days after sowing, seedlings were transplanted into 13 L hydroponic tanks in a glasshouse at the University of Pisa (Pisa, Italy) at a plant density of 720 plants m^−2^. The experiment was conducted in autumn; lasting for 17 days DAP. In the glasshouse, average air temperature was 22.6 °C, with minimum and maximum temperatures of 17.9 °C and 31.5 °C, respectively; natural PAR averaged 8.50 mol m^−2^ d^−1^ and HPS lamps provided 100 µmol m^−2^ s^−1^ PAR for 12 h daily; therefore, total PAR was approximately 12.82 mol m^−2^ d^−1^.

### 4.2. Experimental Design and Nutrient Solutions

Four different nutrient solutions, varying in the concentration of NaCl (0 and 428 mM, which corresponds to 25 g L^−1^) and NO_3_^−^ (10 and 1 mM), were compared in a completely randomized design with three replicates. Each replicate consisted of one hydroponic tank containing 30 individual plants. The mineral composition and electrical conductivity of the nutrient solutions are detailed in Table 4. The saline nutrient solutions were prepared using technical-grade salts—macronutrients from Haifa Chemicals Ltd. (Matam-Haifa, Haifa, Israel) and micronutrients from Vialca Srl (Uzzano, Pistoia, Italy)—dissolved in tap water: 5[Ca(NO_3_)_2_ · H_2_O]NH_4_NO_3_, KH_2_PO_4_, MgSO_4_ · 7H_2_O, KNO_3_, K_2_SO_4_, Fe EDDHA, boric acid, Cu EDTA, Zn EDTA, chelated Mn, Na_2_MoO_4_. Sodium chloride was added gradually over three consecutive days to prevent osmotic shock.

### 4.3. Determinations

#### 4.3.1. Growth Analysis

Two samplings were performed, 10 and 17 days after the onset of the experiment (12 and 19 DAP). Stem and leaf FW and DW, root DW, and leaf area were determined in 18 plants collected from each tank. Dry weight was measured after drying fresh samples in a ventilated oven at 70 °C until constant weight. Leaf moisture content was calculated as the percentage of water in the fresh tissue. Leaf succulence was calculated as the amount of water per unit leaf area. Fresh yield corresponded to shoot FW. A digital planimeter was used to measure the leaf area, and LAI was calculated as individual plant leaf area multiplied by plant density. The leaf and whole-plant DW, and leaf area measured at the beginning of the experiment and 10 and 17 days later were used to calculate the following growth parameters [65]: RGR (g d^−1^), NAR (g m^−2^ d^−1^), LAR (m^−2^ g^−1^), SLA (m^−2^ g^−1^), and LWR (dimensionless). The equations are shown in Table S4.

#### 4.3.2. Mineral Elements

The concentration of mineral elements and NO_3_^−^ was determined in ground dry leaf samples. Samples were either mineralized with a mixture (5:2) of 65% HNO_3_ and 30% H_2_O_2_ at 240 °C for 1 h or extracted with distilled water at room temperature for 2 h. In the mineralized samples, K, Ca, Mg, Na, Cu, Fe, Mn, and Zn were quantified using atomic absorption spectroscopy (Varian Model Spectra AA240 FS, Agilent Technologies Australia [M]. Pty Ltd., Mulgrave, Australia), while P was measured by UV/VIS spectrometry using Olsen’s method. The NO_3_^−^ concentration was analyzed spectrophotometrically in leaf water extracts using the salicylic sulfuric acid method as described by Puccinelli et al. [43]. Dry leaf samples were also used to determine organic N via the Kjeldahl method.

#### 4.3.3. Plant Secondary Metabolites and Antioxidant Capacity

The leaf concentration of pigments, flavonoids, and phenols, and the total antioxidant capacity were analyzed in fresh samples (leaves from four plants per tank). Fresh samples were extracted with 99% (*v*/*v*) methanol, sonicated for 60 min, and then stored at −18 °C for 24 h; afterward, the concentration of total chlorophylls, carotenoids, phenols and flavonoids, and the antioxidant capacity (FRAP assay) were determined spectrophotometrically as reported by Puccinelli et al. [43].

To measure betalain concentration, 100 mg of fresh baby greens were extracted using 50% (*v*/*v*) ethanol [66]. The resulting extract was then diluted with 100 mM phosphate buffer (pH 6.5), and its absorbance was recorded at 538 nm, 476 nm, and 600 nm [67]. Betacyanin and betaxanthin concentrations were calculated [68] and expressed as mg per g of FW. Total betalains were the sum of betacyanins and betaxanthins.

For DPPH radical scavenging activity, 0.1 mL of methanolic extract was mixed with 2.9 mL of DPPH solution (20 mg L^−1^ in methanol), incubated in the dark for 45 min at room temperature, and absorbance measured at 515 nm. Antioxidant capacity was expressed as Trolox equivalent antioxidant capacity, using a Trolox calibration curve [69].

For the determination of ascorbic acid, 500 mg of leaf samples were stored at −80 °C, then extracted with 5 mL of a cold of 50 mM KH_2_PO_4_ solution (pH 7.0) under dim light conditions. The extracts were filtered using Chromafil^®^ Xtra 20/25 H-PTFE syringe filters (Macherey–Nagel, Duren, Germany) and analyzed without further treatment. For the determination of total ascorbic acid, which is the sum of ascorbic acid and dehydroascorbic acids, 50 µL of 2 mM dithiothreitol (DTT, pH 7.0), were added to 500 µL of filtered extract and the solution was stored 20 min in the dark at room temperature before HPLC analysis. Dehydroascorbic acid was determined by the difference between total ascorbic acid and ascorbic acid.

The concentration of total oxalate was measured in dried leaf samples extracted with 0.25 M HCl (50 mg DW in 6 mL) at 100 °C for 30 min. The mixture was allowed to cool, filled to a volume of 10 mL with 0.25 M HCl, and then filtered through filter paper. The soluble oxalate content in each leaf sample was determined as above, using distilled water instead of 0.25 M HCl.

Both ascorbic acid and oxalate were determined with a HPLC-DAD system (Jasco, Tokyo, Japan) consisting of a PU-2089 four-solvent low-pressure gradient pump and a MD-4010 diode array detector. The separation was performed using a C18 Atlantis^®^ T3 5 µm 4.6 × 250 mm column (Waters, Milford, MA, USA). Ascorbic acid and oxalate were determined using 50 mM KH_2_PO_4_ (pH 2.8) in isocratic mode with a flow of 0.8 mL min^−1^, followed by 5 min washing with 95% acetonitrile (CH_3_CN) and 5 min column re-equilibration. The injection volume was 20 µL and the chromatograms were recorded at 243 or 214 nm for ascorbic acid and oxalate, respectively. Ascorbic acid or oxalate standard solutions were used for calibration.

#### 4.3.4. Oxalate Oxidase

Crude extracts were obtained by homogenizing fresh samples in 0.4 M phosphate buffer (pH 7.0), followed by centrifugation. Assays were performed in foil-wrapped tubes containing succinate buffer (0.05 M, pH 5.0), CuSO_4_ (0.01 M), and oxalic acid (0.01 M), incubated at 40 °C for 5 min. A 4-aminophenazone reagent was added, and color developed after 30 min in darkness. Absorbance was measured at 520 nm against a standard H_2_O_2_ curve. One unit of OxO was defined as the amount of enzyme producing 1 µmol of H_2_O_2_ per 5 min under assay conditions [70]. Specific activity was expressed as µmol H_2_O_2_ mg^−1^ protein, with protein determined by using the Bradford method with bovine serum albumin standards [71].

#### 4.3.5. Chlorophyll *a* Fluorescence

Chlorophyll *a* fluorescence transients were determined in dark-adapted leaves maintained for 30 min at room temperature, using a portable Handy PEA fluorimeter (Hansatech, UK). Measurements were taken on the leaf surface (4 mm diameter) exposed to an excitation light of 3000 μmol m^−2^ s^−1^ (600 W m^−2^) emitted by three ultrabright red LEDs with a peak at 650 nm. Leaf fluorescence detection was measured by a fast-response PIN photodiode with an RG9 long-pass filter (Hansatech, technical manual). The JIP test was performed to determine the PI, which integrates several parameters related to photosynthesis and provides an integrative parameter of leaf functionality and vitality [72].

Leaf color variations were determined by measuring CIELAB color space coordinates L*, a*, b* with a Konica Minolta CR-400 colorimeter with D65 illuminant (Konica Minolta Sensing, Inc., Osaka, Japan). The colorimeter was automatically calibrated with an internal standard before each measurement. Lightness was measured directly while HUE angle and Chroma Index were derived from a* and b* values according to Hirschler et al. [73] and Nambi et al. [74].

#### 4.3.6. Ethylene Evolution

Ethylene production was measured by enclosing approximately 1 g of intact fresh leaves in airtight containers (50 mL). Two mL gas samples were taken from the headspace of the containers after 1 h incubation at room temperature. The ethylene concentration was measured with a gas chromatograph (HP 8890) using a flame ionization detector (FID), a stainless-steel column (150 × 0.4 cm ø packed with Hysep T) (Hewlett-Packard, Menlo Park, CA, USA), column and detector temperatures of 70° and 350 °C, respectively, and helium as carrier gas at a flow rate of 30 mL min^−1^. Quantification was performed against an external standard and results were expressed as nL h^−1^ g^−1^ FW.

### 4.4. Statistical Analysis

Data were analyzed with JMP Pro 17 (SAS Institute, Cary, NC, USA). Normality was tested using the Shapiro–Wilk test and homogeneity of variances with Levene’s test. Data were then subjected to two-way ANOVA, with NaCl and NO_3_^−^ concentration as independent variables. Means were compared using Tukey’s HSD test (*p* < 0.05). Percent ratios of soluble to total oxalate were arcsine-transformed prior to analysis but are presented in tables as untransformed values.

## 5. Conclusions

In conclusion, the hydroponic production of red orache baby greens was markedly reduced when grown with solutions containing the typical salinity and nitrogen levels of marine aquaculture effluents. Such solutions should therefore be diluted with freshwater or supplemented with a standard nutrient solution before use. High salinity negatively affected plant growth and leaf quality to a much greater extent than reduced NO_3_^−^ availability. From a nutraceutical perspective, high salinity reduced the leaf concentrations of pigments and flavonoids, as well as total antioxidant capacity. Leaf sodium levels increased under saline conditions but remained within safe limits for moderate daily consumption, i.e., less than 160 g day^−1^, of red orache baby leaves. Both high salinity and low nitrogen supply led to decreased leaf NO_3_^−^ concentrations. High salinity increased leaf oxalate levels, whereas nitrogen availability had only minor effects. Overall, red orache leaves were high in oxalate and should therefore be consumed in moderation. The pattern of oxalate accumulation observed supports the hypothesis that increased oxalate levels are linked to reduced catabolic activity of OxO. To our knowledge, this is the first report of OxO activity in a dicot species.

Future research should focus on developing cultivation strategies to lower oxalate concentrations in hydroponically grown red orache. However, as this study was conducted under controlled hydroponic conditions for a limited growth period, further research is needed to confirm the long-term performance of red orache in diverse saline cultivation systems.

## Figures and Tables

**Table 1:** Leaf fresh weight (FW), leaf, stem, root and total dry weight (DW), leaf area index, leaf moisture content and leaf succulence in red orache (*Atriplex hortensis* var. *rubra*) plants grown hydroponically for 17 days with different nutrient solutions, varying in the concentration of NaCl and NO_3_^−^.

NaCl (mM)	NO_3_^−^ (mM)	Leaf FW (g m^−2^)	Leaf DW (g m^−2^)	Stem DW (g m^−2^)	Root DW (g m^−2^)	Total DW (g m^−2^)	Leaf Area Index	Leaf Moisture Content (%)	Leaf Succulence (kg m^−2^)
0	10.0	1908.0 ± 231.1	124.2 ± 14.3	22.68 ± 2.68	10.10 ± 1.49	157.0 ± 18.3	6.724 ± 0.638	93.4 ± 0.2	0.264 ± 0.011
	1.0	1434.0 ± 30.8	98.2 ± 5.4	17.82 ± 0.69	11.14 ± 0.71	127.2 ± 5.4	5.064 ± 0.128	93.2 ± 0.4	0.264 ± 0.013
428	10.0	500.9 ± 13.6	46.3 ± 2.5	8.24 ± 0.79	2.57 ± 0.30	57.1 ± 3.6	1.152 ± 0.062	90.5 ± 0.4	0.396 ± 0.016
	1.0	345.7 ± 16.8	31.7 ± 1.6	6.40 ± 0.50	2.06 ± 0.58	40.1 ± 2.6	0.756 ± 0.067	90.3 ± 0.2	0.418 ± 0.017
MAIN EFFECT									
0		1671.0 ± 148.7 a	111.2 ± 9.0 a	20.25 ± 1.65 a	10.62 ± 0.78 a	142.1 ± 10.8 a	5.894 ± 0.471 a	93.3 ± 0.2 a	0.264 ± 0.008 b
428		423.3 ± 36.0 b	39.0 ± 3.5 b	7.32 ± 0.59 b	2.31 ± 0.31 b	48.6 ± 4.3 b	0.954 ± 0.097 b	90.4 ± 0.2 b	0.407 ± 0.011 a
	10.0	1204.4 ± 331.2 a	85.2 ± 18.6 a	15.46 ± 3.46	6.33 ± 1.82	107.0 ± 23.8 a	3.938 ± 1.278 a	91.9 ± 0.7	0.330 ± 0.031
	1.0	889.8 ± 243.9 b	64.9 ± 15.1 b	12.11 ± 2.58	6.60 ± 2.07	83.7 ± 19.6 b	2.910 ± 0.965 b	91.7 ± 0.7	0.341 ± 0.036
ANOVA									
NaCl		***	***	***	***	***	***	***	***
NO_3_^−^		*	*	ns	ns	*	*	ns	ns
NaCl × NO_3_^−^		ns	ns	ns	ns	ns	ns	ns	ns

Mean values (n = 3; ±SE) flanked by the same letter, within the same column, are not statistically different at 5% level after Tukey’s post hoc test. *** *p* ≤ 0.001; * *p* ≤ 0.05; ns = not significant.

**Table 2:** Leaf concentration of total phenols and flavonoids, ascorbic acid, and antioxidant capacity measured by Ferric Reducing Antioxidant Power (FRAP) and 2,2-difenil-1-picrilidrazile (DPPH) assay in red orache (*Atriplex hortensis* var. *rubra*) plants grown hydroponically for 17 days with different nutrient solutions, varying in the concentration of NaCl and NO_3_^−^.

NaCl (mM)	NO_3_^−^ (mM)	Phenols (g kg^−1^ DW)	Flavonoids (g kg^−1^ DW)	Ascorbic Acid (g kg^−1^ DW)	Total Ascorbic Acid (g kg^−1^ DW)	FRAP Index (mmol Fe (II) kg^−1^ DW)	DPPH (mmol TE kg^−1^ DW)
0	10.0	16.42 ± 1.93	5.90 ± 0.18	2.52 ± 0.17	3.62 ± 0.13	198.18 ± 16.18	34.99 ± 0.71
	1.0	17.98 ± 1.28	6.37 ± 0.34	2.72 ± 0.15	3.27 ± 0.15	214.48 ± 7.69	34.84 ± 2.48
428	10.0	9.78 ± 0.83	3.12 ± 0.03	1.88 ± 0.27	2.94 ± 0.11	93.86 ± 3.05	16.32 ± 0.57
	1.0	9.35 ± 1.13	2.65 ± 0.08	1.41 ± 0.12	3.16 ± 0.11	96.10 ± 2.82	18.10 ± 0.89
MAIN EFFECT							
0		17.20 ± 1.20 a	6.13 ± 0.22 a	2.62 ± 0.12 a	3.48 ± 0.12	206.33 ± 9.55 a	34.91 ± 1.29 a
428		9.56 ± 0.71 b	2.88 ± 0.11 b	1.65 ± 0.18 b	3.05 ± 0.09	94.98 ± 2.13 b	17.21 ± 0.64 b
	10.0	13.10 ± 1.72	4.51 ± 0.57	2.26 ± 0.20	3.28 ± 0.16	146.02 ± 22.83	25.65 ± 3.84
	1.0	13.66 ± 1.96	4.51 ± 0.78	2.20 ± 0.28	3.21 ± 0.10	155.29 ± 24.51	26.47 ± 3.66
ANOVA							
NaCl		**	***	*	ns	***	***
NO_3_^−^		ns	ns	ns	ns	ns	ns
NaCl × NO_3_^−^		ns	ns	ns	ns	ns	ns

Mean values (n = 3; ±SE) flanked by the same letter, within the same column, are not statistically different at 5% level after Tukey’s post hoc test. *** *p* ≤ 0.001; ** *p* ≤ 0.01; * *p* ≤ 0.05; ns = not significant.

**Table 3:** Leaf concentration of total chlorophylls, carotenoids and betalains, and leaf color parameters in red orache (*Atriplex hortensis* var. *rubra*) plants grown hydroponically for 17 days with different nutrient solutions, varying in the concentration of NaCl and NO_3_^−^.

NaCl (mM)	NO_3_^−^ (mM)	Chlorophylls (g kg^−1^ DW)	Carotenoids (g kg^−1^ DW)	Betalains (g kg^−1^ DW)	Lightness	Chroma	Hue Angles
0	10.0	17.76 ± 0.74	4.21 ± 0.15	5.47 ± 0.41	26.3 ± 0.7	7.76 ± 0.62	62.1 ± 1.4
	1.0	17.08 ± 1.57	4.27 ± 0.36	5.45 ± 0.44	26.4 ± 0.6	7.38 ± 0.98	59.0 ± 5.9
428	10.0	9.38 ± 0.78	1.95 ± 0.15	2.31 ± 0.16	31.4 ± 0.4	7.11 ± 0.26	52.2 ± 7.6
	1.0	8.37 ± 0.19	1.81 ± 0.03	2.06 ± 0.20	30.8 ± 0.4	7.46 ± 0.48	54.8 ± 6.1
MAIN EFFECT							
0		17.42 ± 0.88 a	4.24 ± 0.20 a	5.46 ± 0.30 a	26.3 ± 0.4 b	7.57 ± 0.53	60.5 ± 2.8
428		8.87 ± 0.45 b	1.88 ± 0.08 b	2.18 ± 0.14 b	31.1 ± 0.3 a	7.29 ± 0.26	53.5 ± 4.4
	10.0	13.57 ± 1.79	3.08 ± 0.47	3.89 ± 0.68	28.8 ± 1.2	7.44 ± 0.33	57.2 ± 4.12
	1.0	12.72 ± 1.95	3.04 ± 0.53	3.76 ± 0.73	28.6 ± 1.0	7.42 ± 0.49	56.9 ± 3.9
ANOVA							
NaCl		***	***	***	***	ns	ns
NO_3_^−^		ns	ns	ns	ns	ns	ns
NaCl × NO_3_^−^		ns	ns	ns	ns	ns	ns

Mean values (n = 3; ±SE) flanked by the same, within the same column, letter are not statistically different at 5% level after Tukey’s post hoc test. *** *p* ≤ 0.001; ns = not significant.

**Table 4:** Mineral composition and electrical conductivity of the different nutrient solutions used in the experiment with red orache plants grown hydroponically in a glasshouse.

	Nutrient Solution			
	0 mM NaCL 10 mM NO_3_^−^	0 mM NaCl 1 mM NO_3_^−^	428 mM NaCl 10 mM NO_3_^−^	428 mM NaCl 1 mM NO_3_^−^
NaCl (g L^−1^)	0	25.0	0	25.0
N-NO_3_ (mM)	10.0	1.0	10.0	1.0
P-PO_4_ (mM)	1.5	1.5	1.5	1.5
K (mM)	9.0	8.0	9.0	8.0
Ca (mM)	4.5	4.5	4.5	4.5
Mg (mM)	2.0	2.0	2.0	2.0
S-SO_4_ (mM)	0.7	0.7	7.0	9.0
Cl	0.7	0.7	428.7	428.7
Na (mM)	0.8	0.8	428.8	428.8
Fe (µM)	40.0	40.0	40.0	40.0
B (µM)	40.0	40.0	40.0	40.0
Cu (µM)	3.0	3.0	3.0	3.0
Zn (µM)	10.0	10.0	10.0	10.0
Mn (µM)	10.0	10.0	10.0	10.0
Mo (µM)	1.0	1.0	1.0	1.0
EC (mS cm^−1^)	2.4	2.3	40.1	39.8

## Data Availability

The original contributions presented in this study are included in the article/Supplementary Material. Further inquiries can be directed to the corresponding author.

## Supplementary Materials

The following supporting information can be downloaded at: https://www.mdpi.com/article/10.3390/plants14213292/s1, Figure S1. Leaf performance index in red orache L. plants grown hydroponically for 17 days with different nutrient solutions, varying in the concentration of NaCl and nitrate; Table S1. Relative growth rate (RGR), net assimilation rate (NAR), leaf area ratio (LAR), specific leaf area (SLA), and leaf weight ratio (LWR) in red orache plants grown hydroponically for 17 days with different nutrient solutions, varying in the concentration of NaCl and NO_3_^−^. Mean values (n = 6; ±SE) of the parameters calculated for the first and second growth phase, which ended 10 and 17 days after the onset of the experiment; Table S2. Leaf mineral concentration (on dry weight basis) in red orache L. plants grown hydroponically for 17 days with different nutrient solutions for total salinity and nitrate concentration; Table S3. Adequate ranges for the leaf concentration of macronutrients and micronutrients in *Beta vulgaris* var. *vulgaris* and *Spinacia oleracea*; Table S4. Equations used to calculate the growth parameters of red orache plants grown hydroponically for 17 days with different nutrient solutions, varying in the concentration of NaCl and NO_3_^−^. The parameters were calculated based on the leaf (L) and whole-plant (W) dry weight (g), and leaf area (A, m^2^) measured at the beginning of the experiment and 10 and 17 days later; Table S5. Table of abbreviations.
