# Supplementary material for: Salinity and Nitrogen Availability Affect Growth, Oxalate Metabolism, and Nutritional Quality in Red Orache Baby Greens
Source: Plants (Basel). 2025 Oct 28;14(21):3292. doi: 10.3390/plants14213292 (PMC12610718; doi:10.3390/plants14213292)
Supplement: Supplementary file 1 [file plants-14-03292-s001.zip › plants-3897092-supplementary.pdf]

Supplementary material

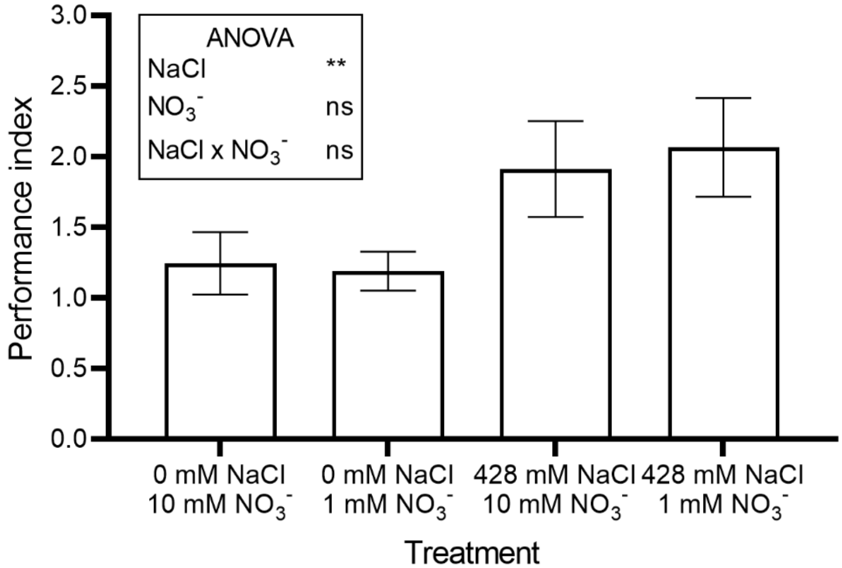

Figure S1. Leaf performance index in red orache L. plants grown hydroponically for 17 days with different nutrient solutions, varying in the concentration of NaCl and nitrate.

Table S4. The equations used to calculate the growth parameters of red orache plants grown hydroponically for 17 days with different nutrient solutions, varying in the concentration of NaCl and NO_3^-. The parameters were calculated based on the leaf (L) and whole-plant (W) dry weight (g), and leaf area (A, m^2) measured at the beginning of the experiment and 10 and 17 days later.

Parameter	Equation
Relative growth rate (RGR; g d^{-1})	$RGR = \frac{(\ln W_2 - \ln W_1)}{t_2 - t_1} = NAR \cdot LAR$
Net assimilation rate (NAR; g m^{-2} d^{-1})	$NAR = \frac{(W_2 - W_1)}{A_2 - A_1} \cdot \frac{(\ln A_2 - \ln A_1)}{t_2 - t_1}$
Leaf area ratio (LAR; m^{-2} g^{-1})	$LAR = \frac{(A_2 - A_1)}{(\ln A_2 - \ln A_1)} \cdot \frac{(\ln W_2 - \ln W_1)}{W_2 - W_1} = SLA \cdot LWR$
Specific leaf area (SLA; m^{-2} g^{-1})	$SLA = \frac{(A_2 - A_1)}{(\ln A_2 - \ln A_1)} \cdot \frac{(\ln W_2 - \ln W_1)}{W_2 - W_1}$
Leaf weight ratio (LWR; dimensionless)	$LWR = \frac{(L_2 - L_1)}{(\ln L_2 - \ln L_1)} \cdot \frac{(\ln W_2 - \ln W_1)}{W_2 - W_1}$

Table S1. Relative growth rate (RGR), net assimilation rate (NAR), leaf area ratio (LAR), specific leaf area (SLA), and leaf weight ratio (LWR) in red orache plants grown hydroponically for 17 days with different nutrient solutions, varying in the concentration of NaCl and NO_3^-. Mean values (n = 6; ± SE) of the parameters calculated for the first and second growth phase, which ended 10 and 17 days after the onset of the experiment.

	RGR (d^{-1})	NAR (g m^{-2} d^{-1})	LAR (m^{-2} g^{-1})	SLA (m^{-2} g^{-1})	LAR
NaCl concentration (mM)					
0	0.241±0.012	5.59±0.379	0.043±0.001	0.053±0.001	0.808±0.011
428	0.173±0.017	7.204±0.366	0.024±0.002	0.029±0.002	0.815±0.009
ANOVA	***	***	***	***	ns
NO_3^- concentration (mM)					
10	0.213±0.021	6.163±0.444	0.035±0.003	0.042±0.004	0.827±0.008
1	0.201±0.02	6.631±0.545	0.032±0.004	0.040±0.006	0.796±0.008
	ns	ns	*	ns	***

*** $P \leq 0.001$; ** $P \leq 0.01$; * $P \leq 0.05$; ns = not significant.

Table S2. Leaf mineral concentration (on dry weight basis) in red orache L. plants grown hydroponically for 17 days with different nutrient solutions for total salinity and nitrate concentration.

NaCl (mM)	NO_3^- (mM)	N-total (g kg^{-1})	P (g kg^{-1})	K (g kg^{-1})	Ca (g kg^{-1})	Mg (g kg^{-1})	Na (g kg^{-1})	Fe (mg kg^{-1})	Mn (mg kg^{-1})	Zn (mg kg^{-1})	Cu (mg kg^{-1})
0	10.0	75.8±1.95	6.67±0.18 b	80.1±1.4	7.263±0.303 a	9.733±0.109	42.5±0.3 d	200.9±5.6	67.3±1.7	79.5±8.5 ab	30.9±8.3
	1.0	66.0±2.15	7.93±0.06 a	73.9±5.2	5.326±0.196 b	9.391±0.359	51.8±1.8 c	148.7±29.0	81.8±2.5	85.2±0.6 ab	29.5±6.6
428	10.0	48.3±2.03	6.58±0.13 b	23.3±0.5	0.571±0.024 b	4.807±0.311	117.4±2.0 b	193.7±26.8	48.1±2.8	63.7±3.3 b	19.0±1.3
	1.0	42.6±2.26	5.38±0.06 c	21.9±0.9	1.162±0.053 b	4.528±0.067	136.0±2.6 a	113.0±8.8	61.8±3.2	92.4±3.1 a	21.3±0.6
MAIN EFFECT											
0		70.9±3.59 a	7.30±0.30 a	77.0±2.8 a	6.294±0.462 a	9.562±0.184 a	47.2±2.2 b	174.8±17.6	74.5±3.5 a	82.4±4.0	30.2±4.7
428		45.4±2.65 b	5.98±0.28 b	22.6±0.6 b	0.866±0.135 b	4.667±0.155 b	126.7±4.4 a	153.3±22.0	54.9±3.6 b	78.1±6.7	20.1±0.8
	10.0	62.0±8.86	6.62±0.10	51.7±12.7	3.917±1.503 a	7.270±1.111	80.0±16.8 b	197.3±12.3 a	57.7±4.5 b	71.6±5.4 b	25.0±4.6
	1.0	54.3±7.67	6.66±0.57	47.9±11.9	3.244±0.936 b	6.959±1.099	93.9±18.9 a	130.8±15.7 b	71.8±4.8 a	88.8±2.1 a	25.4±2.1
ANOVA											
NaCl		***	***	***	***	***	***	ns	***	ns	ns
NO_3^-		ns	ns	ns	**	ns	***	*	***	**	ns
NaCl x NO_3^-		ns	***	ns	***	ns	*	ns	ns	*	ns

Mean values (n = 3; ± SE) flanked by the same letter are not statistically different at 5% level after the Tukey's post-hoc test. *** P ≤ 0.001; ** P ≤ 0.01; * P ≤ 0.05; ns = not significant

Table S3. Adequate ranges for the leaf concentration of macronutrients and micronutrients in *Beta vulgaris* var. *vulgaris* and *Spinacia oleracea*.

	N (g kg^{-1})	P (g kg^{-1})	K (g kg^{-1})	Ca (g kg^{-1})	Mg (g kg^{-1})	Fe (mg kg^{-1})	Mn (mg kg^{-1})	Zn (mg kg^{-1})	Cu (mg kg^{-1})
Table beet	26–40	2–3	17–40	15–30	3–10	40–200	70–200	15–30	5–10
Spinach	15–40	2–5	30–40	6–10	10–16	-	30–50	50–70	5–7

Source: (Hochmuth et al., 2022). "Plant Tissue Analysis and Interpretation for Vegetable Crops in Florida: HS964 EP081 Rev. 11 2022". EDIS 2022 (6). Gainesville, FL. https://doi.org/10.32473/edis-ep081-2004.

Table S5. Table of abbreviations

Extended name	Abbreviation
nitrate	NO_3^-
ascorbic acid	AsA
oxalate oxidase	OxO
ammonium	NH_4^+
fresh weight	FW
dry weight	DW
leaf area index	LAI
relative growth rate	RGR
leaf area ratio	LAR
specific leaf area	SLA
net assimilation rate	NAR
Ferric Reducing Antioxidant Power	FRAP
2,2-difenil-1-picrilidrazile	DPPH
days after planting	DAP
performance index	PI
European Union	EU
photosynthetically active radiation	PAR
